# Videoconference Anxiety: Conceptualization, Scale Development and Preliminary Validation

**DOI:** 10.1192/j.eurpsy.2022.1082

**Published:** 2022-09-01

**Authors:** N. Gözpınar, V. Görmez

**Affiliations:** Göztepe Prof Dr Süleyman Yalçın City Hospital, Child And Adolescent Psychiatry, Istanbul, Turkey

**Keywords:** Children, adolescent, videoconference anxiety, social anxiety disorder

## Abstract

**Introduction:**

With measures of COVID-19, activities that cover a large part of life have started to be carried out via videoconferencing. Videoconferencing can be disadvantageous for individuals with social anxiety due to increased social presence, decreased mutual understanding and consequently causing awkward communication.

**Objectives:**

This study aims to develop a scale to explore the difficulties experienced by individuals with social anxiety during videoconferencing.

**Methods:**

598 children and adolescents between the ages of 11-18 participated in the study. The data were collected with Sociodemographic Information Form, Videoconference Anxiety Scale and Liebowitz Social Anxiety Scale.

**Results:**

According to correlation analysis, all correlations between Videoconference Anxiety Scale and Liebowitz Social Anxiety Scale total score and subscale scores are above 0.50. According to EFA, the scale consisted of 25 items and a single factor. Factor loads were between 0.62 and 0.81, the single factor explained 52.95% of the variance. Model fit indices after CFA were as follows: X2/df:3.360, GFI:.850, IFI:.900, TLI:.890, CFI:.900, RMSEA:.078, SRMR:.0475. Convergent and discriminative validity of the scale was tested. Standardized factor loads of all items were higher than 0.50. AVE value was 0.47, while CR value was 0.96. Cronbach’s alpha coefficient of 25-item VAS is 0.96.

**Conclusions:**

This study showed that Videoconference Anxiety is a phenomen which is higly correlated with social anxiety and Videoconference Anxiety Scale is a valid and reliable instrument for Turkish children and adolescents.

**Disclosure:**

No significant relationships.

